# Examination of referral source and retention among women in residential substance use disorder treatment: a prospective follow-up study

**DOI:** 10.1186/s13011-021-00357-y

**Published:** 2021-03-02

**Authors:** Dean Rivera, Donna Dueker, Hortensia Amaro

**Affiliations:** 1 Suzanne Dworak-Peck School of Social Work, University of Southern California, 669 W. 34th Street, MRF 214, Los Angeles, CA 90089 USA; 2 Department of Health Sciences, California State University, Dominguez Hills, 1000 East Victoria Street, Carson, CA 90747 USA; 3 Herbert Wertheim College of Medicine and Robert Stempel College of Public Health and Social Work, Florida International University, 11200 SW 8th Street, AHC 5, Miami, FL 33199 USA

**Keywords:** Substance use disorder, Mandated residential treatment, Treatment retention, Women, Co-occurring disorder

## Abstract

**Background:**

Court-mandated substance use disorder (SUD) treatment, as compared to nonmandated treatment, has been associated with increased retention and completion. However, whether child protective services (CPS)-mandated women’s residential SUD treatment leads to improved treatment retention in comparison to criminal justice (CJ)-mandated and nonmandated treatment remains unclear.

**Purpose:**

This study compared the number of days retained in residential SUD treatment among three referral sources (CPS, CJ, and nonmandated), while also examining whether having a co-occurring mental health disorder or increased stress, depression, anxiety, and PTSD symptomology contributed to decreased retention. This study tested the hypothesis that women mandated by the CPS and CJ systems would have improved residential SUD treatment retention compared with nonmandated women.

**Methods:**

Multiple regression analyses were conducted on data for a diverse sample of 245 women (Hispanic: *N* = 141, Black: *N* = 50, White: *N* = 50) mandated or nonmandated (CJ: *N* = 114, CPS: *N* = 82, nonmandated: *N* = 49) into residential SUD treatment to determine each group’s treatment retention outcomes. *Results:* Women mandated to SUD residential treatment by the CPS system remained in treatment significantly longer (*p* = .046), compared to women not mandated, representing a 34.4% increase in retention. Findings further revealed a corresponding 2.3% decrease in retention (*p* = .048) for each one-unit increase in a patient’s stress score, whereas those with a co-occurring mental health diagnosis had a 43.6% decrease in SUD treatment retention (*p* < .001).

**Conclusions:**

Policy and clinical considerations include (a) increasing case management support and wraparound services that meet the multiple service needs of women who are nonmandated to residential SUD treatment, and (b) incorporating a more nuanced treatment approach that manages mental health disorders and stress symptomology early in treatment when women are most vulnerable to relapse and treatment dropout.

**Trial registration:**

ClinicalTrials.gov Identifier: NCT02977988 (first posted November 30, 2016; last update posted October 7, 2019); U.S. NIH Grant/Contract: 5R01DA038648.

## Introduction

Since 1980, the number of women incarcerated in U.S. prisons and jails has increased by more than 750%, outpacing the increase in male incarceration by more than 50% [1, 2]. This spike in incarceration rates has been largely attributed to substance abuse, wherein more than 60% of women incarcerated in prison are serving sentences for drug-related offenses [3]. Parental substance abuse is also recognized in most states as a leading cause of child maltreatment and neglect [4, 5]. To address these complex societal problems, mandated residential substance use disorder (SUD) treatment, as an external motivator, has become a commonly used treatment engagement strategy in lieu of prosecution or loss of custodial rights by the criminal justice (CJ) and child protective services (CPS) systems, respectively [6, 7].

Although there is some debate about the relative value of external motivation for increasing SUD treatment retention [8], current evidence shows CJ-mandated treatment results in improved treatment retention as compared to nonmandated SUD treatment [9, 10]. Completing a full treatment program is vital, yet retention in residential SUD treatment is one of the major challenges for women [11]. Compared with individuals who have successfully met treatment goals and completed residential treatment, those who do not complete treatment are generally challenged by continued substance use relapses [11] and continued criminal activity [12].

Prior research on mandated SUD treatment has provided inadequate information on its effects on women’s residential SUD treatment retention due to: (a) largely male samples in studies of CJ-mandated treatment focusing primarily on criminal recidivism and lacking investigation of retention among different treatment modalities [13, 14]; (b) few studies examining the effects of CPS-mandated treatment retention among different modalities of SUD treatment [15, 16]; and (c) no prior research comparing residential SUD treatment retention between women who are mandated by the CPS and CJ systems or not mandated. This lack of sex-specific (women only) mandated residential SUD treatment research highlights the importance of investigating the effects of CPS, CJ, or nonmandated referral status on retention in treatment for this vulnerable population [13–16].

Women entering SUD treatment through the CPS and CJ systems generally have more severe SUDs than their male counterparts [17]. Further, in contrast to women entering mixed-sex residential SUD treatment, those entering women’s SUD treatment are more likely to have extensive histories of substance use [18]. CPS- and CJ-mandated residential SUD treatment is a critical intervention for women, although challenges remain regarding this group’s vulnerability to treatment dropout. Challenges associated with increasing susceptibility to treatment dropout include having a co-occurring mental health disorder [19], increased stress [20, 21], and histories of physical and sexual abuse trauma [22]. A large body of research on SUD treatment recognizes women who remain in treatment longer generally have improved treatment recovery outcomes, such as (a) reduction in substance use and long-term abstinence, (b) improved mental health conditions [23], and (c) increased family reunification rates [24]. These findings call attention to the need to examine treatment retention differences in a sociodemographic diverse group of women mandated (CPS and CJ) or nonmandated into women’s residential SUD treatment.

CJ-mandated residential SUD treatment is generally an accepted predictor of improved treatment outcomes and increased retention or completion rates [9, 10]. In a study using data from 461 participants in the Women, Co-occurring Disorders, and Violence Study, findings indicated that women who reported being mandated remained in residential treatment longer and had a lower risk of treatment dropout (35%) compared to those who were nonmandated [25]. A large study investigating the treatment outcomes of women in the Alcohol and Drug Services Study, reflecting 2395 SUD treatment facilities (residential and outpatient), found that treatment completion was higher among women referred by the CJ system [26]. Further, in a recent systematic review evaluating 154 independent drug court evaluations [13], findings indicated lower rates of recidivism among drug court participants relative to nonparticipants (decrease of 12 percentage points), which included drug-related criminal activity and drug use. However, due to the majority of these meta-analyses featured samples composed mostly of men (84%), in addition to methodological limitations, findings are not generalizable to women-only SUD treatment [13].

Because women may contend with different mandating systems to address their SUD, understanding the variability in treatment retention among different referral statuses is an important factor in developing targeted treatment approaches unique to each condition. These separate referral sources function as three distinct external motivational conditions that may contribute to different residential treatment retention outcomes [27]. For example, to maintain child custody rights, women mandated by the CPS system are expected to complete treatment, and noncompletion of treatment can result in child custody being lost. Women who are mandated by the CJ system are required to complete SUD treatment in lieu of criminal prosecution, and completing treatment can lead to criminal charges being dropped or reduced. Alternatively, women who are nonmandated experience external motivation that often comes in the form of family, significant other, or employer pressures to enter SUD treatment [28]. It is important to note that women mandated into treatment by the CJ system, due to overriding jurisdiction, may also have child custody cases, increasing the external motivation to engage in and complete treatment, whereas those who are CPS mandated or nonmandated may not contend with this same risk of “double jeopardy” [15]. Nevertheless, prior research has not adequately investigated the effects of CPS-mandated residential SUD treatment retention in comparison to CJ-mandated and nonmandated treatment [14, 16, 24].

Despite increased research interest in the role of court-mandated SUD treatment on treatment retention, little is known about the effects of CPS-mandated in comparison to CJ- and nonmandated SUD treatment retention [14, 16]. With the continuing expansion of family drug courts in the United States from two in 1994 to 495 in 2018, this is particularly relevant [29]. For instance, participants in family dependency court completed treatment at rates 20 to 30 percentage points higher than that of other parents not mandated by family dependency court, while remaining in SUD treatment longer [24]. Nevertheless, no known studies have examined CPS-mandated women’s SUD residential treatment retention outcomes in comparison to CJ-mandated and nonmandated treatment [14, 24]. Because women enter women’s residential SUD treatment through coercively distinct pathways (CPS, CJ, or nonmandated), it is critical to understand the treatment retention differences among these groups to provide more effective treatment.

Moreover, among women who enter residential SUD treatment, the evidence is not clear whether having a co-occurring mental health disorder is a predictor of decreased treatment retention [19, 30]. Research that has examined associations between co-occurring mental health and SUD treatment has often found that having a mental health disorder is associated with lower treatment retention and poorer outcomes [19, 31]. However, the impact of co-occurring mental health disorders on SUD treatment retention may not always be uniform, varying by treatment modality, psychiatric diagnosis, and sex [19, 30–32]. For example, Choi et al. [30] found women with a co-occurring disorder were more likely to stay longer in treatment when compared to men with a co-occurring disorder. In contrast, prior work has shown women with co-occurring disorders in general have poorer SUD treatment retention than women without a co-occurring disorder [33]. Advancing the current literature, a better understanding of whether having a co-occurring mental health disorder affects SUD treatment retention among mandated (CPS and CJ) or nonmandated women may have implications for women’s SUD treatment, particularly given the distinct coercive pressures associated with the three respective referral conditions.

To gain a fuller understanding and address the aforementioned limitations, this study examined whether increases in psychological symptomology or having a co-occurring mental health diagnosis are contributors to SUD treatment retention among women mandated and nonmandated into residential treatment. These psychological factors include increased levels of stress [20, 34], depression symptomology [35], posttraumatic stress disorder symptomology [36], and anxiety symptomology [37, 38]. However, few studies have examined if these factors affect treatment retention between mandated and nonmandated referral statuses. Therefore, the current study sought to address each of these gaps by comparing samples of women mandated (CPS and CJ) or nonmandated into women’s residential SUD treatment to determine whether having a co-occurring mental health disorder or increased psychological symptomology affected number of days retained in treatment.

### Study hypotheses

Guided by prior empirical research, the primary purpose of this study is to examine differences in women’s residential SUD treatment retention (as measured by number of days in treatment) by referral status (CJ, CPS, and nonmandated). To address these limitations in research, this study used data from women admitted to a residential SUD treatment program who took part in a larger randomized clinical trial [39, 40]. The goal of this study is to generate important insights for understanding retention differences among women entering residential SUD treatment via three primary treatment entry pathways. Moreover, understanding the effects on retention from having a co-occurring mental health disorder and increased PTSD, depression, stress, and anxiety symptomology may aid treatment planning that mitigates early treatment dropout. Hypothesis 1 posits that women mandated by the CPS or CJ system into residential SUD treatment will each be associated with more days retained in treatment compared to those nonmandated. Hypothesis 2 posits that women mandated (CPS and CJ combined) into residential SUD treatment will be associated with more days retained in treatment compared to those who were nonmandated to treatment. Hypothesis 3 posits that women mandated by the CJ system into residential SUD treatment will be associated with more days retained in treatment compared to those mandated by the CPS system. Further, Hypothesis 4 posits increased stress; anxiety, depression and posttraumatic stress symptomology; and having a co-occurring mental health diagnosis will be associated with fewer days retained in treatment.

## Methods

### Study design

Data utilized in this paper were from baseline interviews conducted as part of a randomized controlled trial conducted between 2016 and 2018 (for parent study details, see [40]). In the current study, we did not focus on intervention effects; therefore, we controlled for group assignment in the analysis. The parent study was a parallel-group trial (NCT02977988) designed to compare retention days between women randomly assigned to one of two study conditions during residential SUD treatment: (a) a mindfulness-based intervention and (b) education regarding the neurobiology of addiction, which served as the control group. Baseline interviews occurred prior to randomization and intervention delivery. Randomization into the control and intervention groups occurred via a simple randomization procedure. All participants received SUD treatment as usual in a therapeutic community setting without affecting standard level of care typically provided to patients [39, 40]. Treatment retention data for the study were abstracted from treatment site records upon patients’ discharge from the program and were based on treatment entry date and the date of discharge. The three types of referral status (CJ, CPS, and nonmandated) were likewise obtained from clinical records.

### Study site

The study site was a publicly-funded residential SUD treatment program for women diagnosed with a SUD located in Southern California. The program provides interagency collaborative support services for women referred through the CJ and CPS systems, along with women-specific SUD treatment for those that are self-referred. Alongside SUD treatment, integrated services include mental health counseling, trauma-informed care, and services to those with health problems such as HIV/AIDS. While clients were able to remain in treatment for up to 12 months, the average length of stay was 4 months. The residential SUD treatment program provides dedicated women’s groups, family education and therapy, trauma-informed care, case management, nutritional education and support, and health and wellness programs. The program further provides integrated childcare, resources for children, medical and dental treatment for both women and children, GED courses, an Early Head Start program funded by the federal government, and a preschool program funded by the school district.

### Participants and procedures

All participants (*N* = 245) were adult women (aged 18–65) diagnosed with a SUD, alcohol use disorder (AUD), or both and who spoke English. Women were mandated by the CJ and CPS systems or nonmandated (i.e., self-referred) into residential treatment. Women could leave the program at any time, although those mandated by the CJ or CPS systems could face legal or child custody consequences if the full range of treatment mandated by the CJ or CPS systems was not completed. The facility intake counselor identified clients who were eligible and informed the women about the study. The women who assented were contacted by a study interviewer who made appointments with prospective participants, conducted the informed consent and HIPAA process, and performed the baseline assessment interview. Trained research interviewers using computer-assisted interview procedures collected data from all study participants [39, 40].

### Inclusion and exclusion criteria

Eligibility criteria were as follows: participant housed at SUD program treatment site, female, adult (18–65 years old), able to speak fluent English, diagnosed with a SUD, and signed an informed consent form agreeing to participate in research. Exclusion criteria were as follows: inability to comprehend or sign informed consent to participate due to language barrier, severe cognitive impairment, untreated psychotic disorder, severe mental health condition, past-30-day suicidality based on clinical assessment, currently incarcerated, participating in other research, > 6 months pregnant, > 65 years of age, and not willing to sign HIPAA form or be audio recorded during interviews and intervention sessions [39, 40].

### Data sources and measures

#### Demographic, clinical, referral, and retention data

Baseline demographic information was obtained via in-person interview and included age, race and ethnicity, parental status, housing status, education, and employment status. Admitted patients met one-on-one with a clinician coordinator who assessed for mental health diagnoses, substance or alcohol use disorders, and suicidality using the DSM-5 [41]. Nonmandated patients self-referred at their own initiative or as a result of a recommendation from a nonmandating provider, family member, or friend. For women who were mandated, legal and child custody status was monitored by CJ and CPS systems throughout treatment with updates in addition to coordinated and integrated interagency support and case management services.

#### Self-report measures: depression, anxiety, stress, and post traumatic stress symptomatologies

Psychological symptoms were measured via the Depression, Anxiety and Stress Scale (DASS-21) and the Posttraumatic Symptom Scale-Interview (PSS-I) [42, 43]. The DASS-21 features three self-report subscales designed to measure the emotional states of depression (7 items), anxiety (7 items), and stress (7 items) on a 4-point Likert scale. The PSS-I was utilized to assess frequency of posttraumatic symptom severity in the last 30 days and includes re-experiencing (5 items), avoidance (7 items), and arousal (5 items) on a 4-point Likert scale. Participants replied regarding any traumatic event experienced, not an explicit traumatic event as the traditional instructions recommend.

### Data analysis

Descriptive characteristics of pretreatment factors were examined for the three referral groups (CJ mandated, CPS mandated, and nonmandated). Utilizing *t*-tests and analysis of variance for comparison of means for continuous variables and chi-square tests for comparison of percentages with categorical variables, we compared sociodemographic and pretreatment characteristics among the referral groups. If significant differences between groups were found, pairwise comparisons (i.e., CPS versus CJ, CPS versus nonmandated, CJ versus nonmandated, and mandated versus nonmandated) were conducted. For continuous variables, Tukey’s honestly significant difference method was used to test all possible pairwise differences of means at the same time among the three referral sources (CJ, CPS, and nonmandated). For categorical variables, chi-square tests were conducted for each mandating agency and nonmandated group. If the chi-square *p*-value was less than .017 (i.e., alpha .05 divided by 3) based on number of group comparisons, the difference was considered statistically significant.

All study variables were tested for confirmation in meeting core model assumptions of normality, linearity, and homoscedasticity. A log10 transformation was conducted for the dependent variable, number of days in treatment, to reduce skewness. Additionally, a square-root transformation was conducted for independent variables of age, posttraumatic stress scores, and stress scores to reduce skewness and improve normality.

To address Hypotheses 1, 2, and 3, a multiple linear regression model-building method was used to determine if mandated (CJ or CPS) or nonmandated referral status predicted increased days in treatment. The primary independent variable of interest was referral status, and number of days in treatment was the outcome variable. Univariate analyses were conducted to determine potential associations between being CJ mandated, CPS mandated, or nonmandated and the number of days in treatment.

Next, potential covariates and control variables were evaluated to determine if there was a greater than 10% change in the effect estimate of the mandating variable (CJ or CPS) or nonmandating variable and the number of days in treatment. Based on prior work, potential covariates and control variables tested for possible inclusion in the final models were (a) age, race and ethnicity, being a mother, housing, education, and employment status and (b) number of mental health diagnoses, posttraumatic stress symptomology, depression, anxiety and stress; and type of SUD diagnoses. Control variables determined to be retained in final models were age, stress (symptomolgy), and number of mental health diagnoses.

Addressing Hypothesis 4, all models were examined for stress, having one or more mental health diagnoses, and their association with increased days in treatment among the three treatment referral groups. For all final multiple linear regression models, parameter estimates and corresponding *p*-values are presented. Percentages representing log10 back-transformed parameter estimates are further presented for clarity of interpretation. All statistical analyses were conducted using SAS version 9.4.

## Results

### Participant demographics, mental health, and referral status comparison

Descriptive sociodemographic characteristics are presented in Table 1. The sample featured 245 women; 46.5% were mandated to SUD treatment by the CJ system, 33.5% by the CPS system, and 20% were nonmandated. Ages varied from 18 to 61 years, with a mean of 32.2 years. Most of the women had children (89.8%). The majority of participants were Hispanic (57.6%), followed by non-Hispanic White (20.4%), non-Hispanic Black (20.4%), and another race or ethnicity (1.6%). Most of the participants were homeless or had unstable housing prior to entering treatment (82%), nearly half had less than a high school diploma (47.8%), and many were unemployed (73.9%). More than half of the participants had one or more co-occurring mental health disorders (57.7%) in addition to a SUD diagnosis. In addition, the majority (76.2%) of women had a SUD and 10% had an AUD, whereas 13.8% had both a SUD and AUD.

**Table 1:** Participant Sociodemographic and Pretreatment Characteristics^a^

	CJ	CPS	Nonmandated	Mandated	Total		
	(*N* = 114)	(*N* = 82)	(*N* = 49)	(*N* = 196)	(*N* = 245)		Pairwise^c^
Characteristics	*M* (*SD*) or *N* (%)	*M* (*SD*) or *N* (%)	*M* (*SD*) or *N* (%)	*M (SD)* or *N* (%)	*M* (*SD*) or *N* (%)	Sign. test	*P* group significance
Number of days in treatment	133.86 (79.43)	116.59 (65.59)	96.11 (72.09)	126.61 (74.25)	120.64 (74.67)	*F* = 4.54	.012 CJ/NM; M/NM
Age^e^	32.89 (9.92)	30.33 (6.34)	33.76 (9.53)	31.82 (8.68)	32.21 (8.87)	*F* = 2.97	.053
Race and ethnicity						χ^2^ = 5.7	.458
Non-Hispanic White	19 (16.67)	17 (20.73)	14 (28.57)	36 (18.37)	50 (20.41)		
Non-Hispanic Black	29 (25.44)	13 (15.85)	8 (16.33)	42 (21.43)	50 (20.41)		
Hispanic	64 (56.14)	51 (62.2)	26 (53.06)	115 (58.67)	141 (57.55)		
Other	2 (1.75)	1 (1.22)	1 (2.04)	3 (1.53)	4 (1.63)		
Children						χ^2^ = 10.87	< .001 CJ/CPS; CPS/NM
Yes	97 (85.09)	81 (98.78)	42 (85.71)	178 (90.82)	220 (89.8)		
No	17 (14.91)	1 (1.22)	7 (14.29)	18 (9.18)	25 (10.2)		
Housing						χ^2^ = 1.41	.49
Homeless or housing unstable	97 (85.09)	65 (79.27)	38 (79.17)	162 (82.65)	200 (81.97)		
Stable	17 (14.91)	17 (20.73)	10 (20.83)	34 (17.35)	45 (18.03)		
Education						χ^2^ = 3.95	.41
Less than high school diploma	51 (44.74)	41 (50.00)	25 (51.02)	92 (46.94)	117 (47.76)		
High school diploma or equivalent	33 (28.95)	25 (30.49)	9 (18.37)	58 (29.59)	67 (27.35)		
Some post-high school	30 (26.32)	16 (19.51)	15 (30.61)	46 (23.47)	61 (24.9)		
Employment							
Full-time	11 (9.65)	14 (17.07)	9 (18.37)	25 (12.76)	34 (13.88)		
Part-time	13 (11.40)	8 (9.76)	9 (18.37)	21 (10.71)	30 (12.24)		
Not working	90 (78.95)	60 (73.17)	31 (63.27)	150 (76.53)	181 (73.88)		
Number of mental health diagnoses^b^						χ^2^ = 1.22	.542
None	51 (44.74)	29 (37.18)	21 (44.68)	80 (41.67)	101 (42.26)		
One or more	63 (55.26)	49 (62.82)	26 (55.32)	112 (58.33)	138 (57.74)		
Mental health symptomology^d^							
Posttraumatic stress symptoms^e^	14.96 (12.18)	19.00 (11.99)	21.84 (13.81)	16.65 (12.23)	17.69 (12.71)	*F* = 5.91	.003 CJ/NM; M/NM
Depression symptoms	3.87 (4.00)	5.79 (5.03)	7.47 (6.23)	4.67 (4.55)	5.23 (5.04)	*F* = 10.20	< .001 CJ/NM; CPS/CJ; M/NM
Stress symptoms^e^	5.69 (4.53)	8.05 (4.85)	8.96 (4.51)	6.68 (4.80)	7.13 (4.82)	*F* = 10.90	< .001 CJ/NM; CPS/CJ; M/NM
Anxiety symptoms	4.53 (4.01)	6.26 (4.38)	7.96 (4.76)	5.25 (4.24)	5.79 (4.48)	*F* = 11.69	< .001 CJ/NM; CPS/CJ; M/NM
Substance use disorder diagnosis^b^						χ^2^ = 6.86	< .001
Alcohol use disorder	9 (7.89)	7 (8.97)	8 (17.02)	16 (8.33)	24 (10.04)		
Substance use disorder	86 (75.44)	59 (75.64)	37 (78.72)	145 (75.52)	182 (76.15)		
Both	19 (16.67)	12 (15.38)	2 (4.26)	31 (16.15%)	33 (13.81)		

^a^Table consists of categorical and continuous variables by group (mandating agencies, mandated and nonmandated status). CJ = criminal justice; CPS = child protective services

^b^Frequency missing (*N* = 6); two missing from mandated group and four from nonmandated group for each variable

^c^Pairwise between group significance (CJ = criminal justice; CPS = child protective services; M = mandated; NM = nonmandated)

^d^Mental health characteristics and stress scores reflect symptom severity ranges (higher scores represent increased severity)

^e^Square-root transformations were conducted for age, PTSD, and stress scores to reduce skewness and improve normality

Bivariate analyses identified significant differences by mandated status for being a mother (*p* < .001), PTSD (*p* = .003), depression symptomology (*p* < .001), anxiety symptomology (*p* < .001), perceived stress (*p* < .001), and type of SUD diagnosis (*p* < .001; Table 1). In addition, there was a significant difference between referral status category and number of days retained in treatment (*p* = .012). Pairwise comparisons (uncontrolled) found that women mandated to treatment by either the CJ or CPS system were retained in treatment more days than nonmandated participants. Significant pairwise group comparisons are presented in Table 1.

### Findings for main hypotheses

Tables 2, 3 and 4 present models of separate regressions for the number of days an individual was retained in SUD treatment relative to being CPS, CJ, or nonmandated. Hypothesis 1 (Table 2) was partially confirmed, showing that women mandated by CPS remained in treatment significantly longer (*p* = .046), representing a 34.4% increase in the number of days retained in treatment compared to those who were nonmandated, holding all other variables in the model constant. However, women mandated by the CJ system did not remain in treatment significantly longer (*p* = .056) relative to those that were nonmandated, holding all other variables in the model constant. Addressing Hypothesis 2 (Table 3), analysis revealed being mandated (CPS and CJ groups combined) was a significant predictor of number of days retained in SUD residential treatment (*p* = .032), representing a 32.8% increase compared to those who were nonmandated, holding all other variables in the model constant.

**Table 2:** Multiple Regression Predicting Retention by CJ Mandated and CPS Mandated Compared to Nonmandated^a^

Parameter	*b*	*p*
Intercept	1.77	< .001
Criminal justice^a^	0.12	.056
Child protective services^a^	0.13	.046
Age	0.04	.158
Stress score	−0.01	.048
1 or more mental health diagnoses^b^	−0.16	< .001

Parameter estimates were log10 back-transformed for results section

^a^CJ (*N* = 114); CPS (*N* = 82). Nonmandated (*N* = 49) is reference group

^b^No mental health diagnoses is reference group

**Table 3:** Multiple Regression Predicting Retention by Mandated (CJ and CPS) Compared to Nonmandated^a^

Parameter	*b*	*p*
Intercept	1.77	< .001
Mandated^a^	0.12	.032
Age	0.04	.162
Stress score	−0.01	.046
1 or more mental health diagnoses^b^	−0.16	< .001

Parameter estimates were log10 back-transformed for results section

^a^Mandated (*N* = 196). Nonmandated (*N* = 49) is reference group

^b^No mental health diagnoses is reference group

**Table 4:** Multiple Regression Predicting Retention by CPS Mandated and Nonmandated Compared to CJ Mandated^a^

Parameter	*b*	*p*
Intercept	1.89	< .001
Criminal justice^a^	0.01	.86
Nonmandated^a^	−0.13	.056
Age	0.04	.16
Stress score	−0.01	.048
1 or more mental health diagnoses^b^	−0.16	< .001

Parameter estimates were log10 back-transformed for results section

^a^CJ (*N* = 114); nonmandated (*N* = 49). CPS (*N* = 82) is reference group

^b^No mental health diagnoses is reference group

Hypothesis 3 was not supported (Table 4), showing that women who were mandated by the CJ system did not remain in treatment significantly more days (*p* = .86) compared to those mandated by the CPS system, while controlling for all other variables in the model. Addressing Hypothesis 4, findings in all three models revealed that higher stress scores and having one or more mental health diagnoses were significantly associated with fewer days of SUD residential treatment. Findings revealed that for each one-unit increase in a patient’s stress score, there was a corresponding 2.3% decrease in the number of days an individual was retained in treatment (*p* = .048), holding all variables in the model constant. For women with one or more mental health diagnoses, in addition to an AUD or SUD diagnosis, there was a 43.6% decrease in the number of days they were retained in treatment (*p* < .001), controlling for all other variables in the model.

## Discussion

This is the first known study comparing the associations between women’s residential SUD treatment referral groups (CPS, CJ, and nonmandated) and treatment retention, as measured by the number of days an individual was retained in treatment. Results from this study contribute to the literature on mandated (CJ and CPS) and nonmandated women’s residential SUD treatment. Findings from this study confirm that women mandated into residential SUD treatment by the CPS system, compared to women who were nonmandated, were associated with more days retained in treatment. Contrary to our hypothesis, we did not find that women mandated into residential SUD treatment by the CJ system had significantly improved treatment retention as compared to women mandated by the CPS system or nonmandated. Our study did find that women who had one or more mental health diagnoses, in addition to an AUD or SUD diagnosis, had significantly decreased retention as measured by number of days retained in residential SUD treatment. Further, patients with increased stress was significantly associated with reduced treatment retention.

### Mandated treatment as a predictor of residential SUD treatment retention

Findings provide key insights into the role of three referral conditions with different impacts on treatment retention. These three referral conditions may involve different external motivational factors that may affect treatment retention. For example, our study shows women who entered SUD treatment through mandated referral from the CPS system had improved treatment retention compared to those who were nonmandated. This raises important emergent presuppositions pertaining to the treatment motivation of CPS-mandated and nonmandated women who enter treatment, which may explain these disparate findings [44]. For instance, additional support accompanies women mandated by the CPS system in the form of: (a) cross-system CPS and SUD treatment case management on the level of care needed, treatment planning, and SUD treatment placement; and (b) ongoing monitoring of patient advancement with interagency progress meetings during SUD treatment [44].

Moreover, unlike women who are nonmandated, women mandated by the CPS system typically have extended resources and incentives that are integrated into SUD treatment through case management and childcare supports [44, 45]. Research has demonstrated that interagency (CPS and SUD treatment program) planning and support during SUD treatment encompasses case management and wraparound services that meet multiple service needs of parents with SUDs, including (a) parenting classes or groups, (b) housing transition supports after treatment completion, and (c) matching and obtaining social services tailored to the women and their children’s respective needs. Research has shown that integrated CPS and SUD treatment services lead to improved retention, reductions in substance use, and family reunification [44, 45]. This suggests that combined cross-system supports accompanying the external motivation associated with completion of CPS-mandated treatment (e.g., retaining child custody rights and family reunification) promotes improved treatment retention and outcomes, which is not otherwise available to nonmandated women.

Alternatively, CJ-mandated women who have had several SUD treatment episodes and incarceration experiences or have already lost custody of their children may not have the same external motivation to complete treatment as women who retain partial or full custodial rights and do not have an extensive criminal record, wherein dismissal of current charges upon treatment completion would improve their life circumstances. Although mandated residential SUD treatment, as an external motivation mechanism, is an accepted predictor of improved retention [9, 10, 16], results from this study reveal this may not apply when CJ- and CPS-mandated treatment are considered.

As revealed by the variability in SUD treatment retention between referral groups, future research is needed examining optimal or suboptimal motivational profiles that are associated with retention in residential treatment. More generally, this study demonstrates that investigation and assessment of patient motivation levels prior to and during the treatment episode will provide a more robust understanding of the diverse external motivation-related patient characteristics that promote or thwart residential SUD treatment retention. Additionally, CPS- and CJ-mandated referrals to SUD treatment can occur at various stages of CJ and CPS system involvement (pre- or postadjudication) and therefore, can be associated with different levels of consequences. Consideration by policymakers and clinicians of these factors and their relationships to patient motivation levels and CPS-, CJ-, and nonmandated treatment retention is warranted.

Contrary to our second hypothesis, we did not find that women mandated by the CJ system had significantly increased treatment retention as compared to those mandated by the CPS system. Although a portion of women who entered treatment via the CJ system may have experienced the “double jeopardy” threat of having a CPS case running concurrently, findings suggest this did not induce increased motivation leading to increased treatment retention. For this study, we only had data on primary referral source; therefore, we couldn’t control for any concurrent legal involvement.

Considering the effect of CPS-mandated treatment on retention, it is worth noting that bivariate findings showed a greater percentage of women in the CPS group had children as compared to those in the CJ or nonmandated group (uncontrolled). Such differences may reflect the increased percentage of these women having children, along with an optimal motivation level associated with the mandated requirement for completing treatment to retain child custody rights. Additionally, women who were nonmandated into treatment exhibited increased symptomology scores for mental health characteristics as compared to those who were mandated (CPS and CJ combined) into residential SUD treatment. Such differences may reflect variation in the stages of the recovery process or the level of pretreatment engagement with services, which are likely greater among those who are CPS and CJ mandated to treatment. The variations in clinical profile, stages in recovery process, and level of pretreatment engagement between those mandated and nonmandated into residential SUD treatment are a critical component to be considered in the assessment and treatment planning process to improve treatment retention and outcomes.

### Co-occurring mental health disorders and stress as a predictor of treatment retention

In each model, co-occurring mental health disorders and stress negatively affected the number of days retained in treatment among women from all referral sources. For women with co-occurring disorders, this is consistent with prior evidence showing that patients with higher psychiatric comorbidity receiving SUD treatment have increased odds of treatment noncompletion and earlier attrition rates [19]. Prior work has suggested that individuals with SUDs and psychiatric comorbidities may have more difficulty integrating and fully participating in SUD treatment programs [46, 47]. This is exacerbated when women who enter into residential SUD treatment are challenged with more severe mental health conditions, overloading treatment retention and completion efforts [18, 26, 48]. A greater comprehension of this complex interplay of treatment integration and engagement among women with co-occurring disorders and SUD treatment retention may inform improved design of tailored services for this vulnerable subgroup of women.

Additionally, consistent with prior research, current findings revealed that higher levels of perceived stress were negatively associated with number of days retained in treatment across models [34]. Supporting this finding, prior work has shown that individuals who report higher levels of stress experienced more frequent and stronger substance and alcohol use cravings [21, 49]. Importantly, Law et al. [21] found that craving mediated the relationship between stress and relapse during treatment. This supports the explanation that people with higher perceived stress were negatively associated with number of days retained in treatment, given that relapse is a well-established predictor of treatment dropout [34]. Findings support the need for targeted clinical assessment and observation of women in the early stages of residential treatment given the susceptibility to stress and craving [34], in addition to those with co-occurring mental health conditions. Current clinical and research informed science would benefit by examining the complex relationships between early treatment dropout, co-occurring mental health disorders, and increased stress, which may be differentially mediated by the associations between the three referral groups (CJ, CPS, and nonmandated).

### Limitations

As with any scientific study, there are limitations to the current study. First, findings are limited to a single treatment program and modality of women’s residential SUD treatment. This limits generalizability to other treatment modalities, in addition to mixed-sex or all-male residential SUD treatment programs. Second, CJ- and CPS-mandated women did not differ significantly in the number of days retained in treatment, which may suggest some women in the CPS group may have open CJ cases running concurrently. Third, the current study did not analyze program-level factors associated with early treatment dropout (i.e., insurance limitations for nonmandated women and family financial obligations) [50, 51]. A strength of this study is its rigorous approach to measurement, having drawn data from a randomized controlled trial, in addition to its diverse representativeness of women with CJ and CPS histories with lower socioeconomic status. Thus, findings are likely more generalizable to women served in most publicly funded women’s SUD residential treatment programs with similar demographic characteristics.

## Conclusion

This study provides several implications for women’s mandated residential SUD treatment. The current study demonstrated that women who were CPS mandated into women’s residential SUD treatment had more days retained in treatment compared to nonmandated women. These findings highlight that CPS-mandated treatment, as an external motivator, results in increased treatment retention. These findings indicate that unlike women who are not mandated, those mandated by the CPS system typically have extended resources and incentives that are integrated into SUD treatment through case management and childcare support, promoting treatment engagement and lengthier treatment stays. Moreover, women with co-occurring mental health disorders and increased stress are at a heightened risk of early dropout and reduced residential treatment retention. Clinicians should identify women at treatment entry with co-occurring disorders and increased stress so that individualized intervention approaches can mitigate these risk factors associated with reduced retention.

The current study’s findings highlight the impact that referral source, co-occurring mental health disorders, and stress play as a role in women’s SUD residential treatment retention. This is particularly important given the complex external motivation conditions women face when mandated (CPS or CJ) or nonmandated into SUD treatment, which is further compounded by dynamic intersections of risk associated with having a co-occurring disorder and stress on treatment retention. Clinical implications may include incorporating a more nuanced treatment approach that manages mental health disorders and stress symptomology early in treatment when women are most vulnerable to relapse and treatment dropout. Further, policy considerations should be made which involve increasing case management support and wraparound services that meet the multiple service needs of parents who are nonmandated to residential SUD treatment. This may encompass increased community-based interagency collaboration that addresses the needs of the whole family early in treatment allowing the parent to focus on their treatment while enabling full familial community reintegration at treatment discharge. This study is an important starting point in understanding the complex psychosocial structures related to mandated and nonmandated women’s residential substance abuse treatment and their associations with residential SUD treatment retention.

## Data Availability

The data used or analyzed in the current study are available from Hortensia Amaro, the senior author of this paper and PI of the parent grant, based on available time and resources.

## Abbreviations

CJ: Criminal justice
CPS: Child protective service
SUD: Substance use disorder
AUD: Alcohol use disorder
PTSD: Posttraumatic stress disorder
DASS-21: Depression, Anxiety and Stress Scale
PSS-I: Posttraumatic Symptom Scale-Interview
